# Does an incremental approach to implementing programmatic assessment work? Reflections on the change process

**DOI:** 10.15694/mep.2020.000055.1

**Published:** 2020-03-27

**Authors:** Frank Bate, Sue Fyfe, Dylan Griffiths, Kylie Russell, Chris Skinner, Elina Tor

**Affiliations:** 1 University of Notre Dame Australia; 2 Curtin University

**Keywords:** Assessment, programmatic assessment, learning portfolio.

## Abstract

This article was migrated. The article was marked as recommended.

In 2017, the School of Medicine (Fremantle) of the University of Notre Dame Australia began moving towards programmatic assessment. Programmatic assessment seeks to achieve robust assessment validity through the assessment of a large number of low-stakes activities or data points. These data points exemplify assessment *as* learning by valuing feedback, discussion and reflection, ultimately leading to deeper student engagement without compromising credible decision-making on student progress. The School adopted an incremental approach to implementing programmatic assessment that included first establishing data-informed mentoring, and then activating a continuous assessment program that contributed simultaneously to student learning and School decision-making. Action research helped understand the impact of the initiative. Re-engineering continuous assessment as an incremental step towards programmatic assessment proved to be problematic. Some ideas are proposed to draw the strands of programmatic assessment together that may be useful for others to chart a more fruitful path.

## Introduction

Doctors typically enter the profession by successfully completing a set of high-stakes examinations developed according to stringent psychometric principles. Those administering these examinations usually have confidence in the reliability of the tools and are satisfied that graduates will enter the health system with the requisite knowledge, skills and attitudes to commence their careers as doctors. However, high-stakes examinations tend to generate unnecessary stress amongst students and promote strategic learning ( Ramsden, 1992).

Programmatic assessment has emerged in response to a perceived need to better integrate assessment *for* learning and assessment *of* learning ( van der Vlueten *et al.,* 2012). It is defined as “an integral approach to the design of an assessment program with the intent to optimize its learning function, its decision-making function, and its curriculum quality-assurance function” ( Heeneman *et al,* 2016, p.60). Van Melle *et al.* (2019) argue that programmatic assessment is fundamental to competency-based medical education.

Programmatic assessment rejects the dichotomy between formative and summative assessment, rather, conceptualizing assessment as a continuum of stakes that underpin decision-making with high-stake decisions requiring more data than low-stake decisions. In programmatic assessment, a data point is a piece of information emanating from student interaction with the curriculum. A large number of low-stake data points, systematically collected and acted upon, strengthens the validity of decision-making about student progression. The key principles underpinning programmatic assessment are set out by van der Vlueten *et al.* (2015). Briefly summarizing, programmatic assessment comprises of five principles:

• Continuous: data points are sequenced and embedded as part of the delivery of curriculum to optimize ongoing opportunities for learning.
• Feedback-rich: multiple feedback opportunities are provided to students on all low- and high-stakes data points.
• Mentored: data-informed mentoring encourages students to reflect and plan based on their progress in conjunction with their mentor.
• Coherent: data points are mapped to an overarching structure (e.g. competency framework) so that students understand the value of what they learn.
• Transparent: a systematic approach to collecting and interpreting data points is adopted and explicit processes underpin the trustworthiness of professional judgments.

The School of Medicine Fremantle (the School) of the University of Notre Dame Australia (UNDA) offers a four-year doctor of medicine (MD) degree with about 100 students enrolling each year. The first two years are pre-clinical, consisting of weekly problem-based learning scenarios supported by lectures and small group teaching. The final two years comprise of a series of clinical rotations, primarily located at hospital sites. Each year of the MD is a course that a student needs to pass in order to progress to the next.

The School’s assessment mix currently includes knowledge-based examinations (multiple choice, short answer and case-based), Objective Structured Clinical Examinations, workplace-based assessments and rubric-based assessment activities. Examinations are administered mid-year and end-of-year for pre-clinical students and at the end of the year only for clinical students.

In 2017, the School commenced a journey towards programmatic assessment adopting an incremental approach which initially involved two steps: the establishment of data-informed mentoring (2017) and the re-engineering of the learning portfolio to better integrate continuous assessment into the overall assessment mix (2018). These initiatives were introduced to first year students in 2017 and first and second year students in 2018. Both steps are fundamental to programmatic assessment ( van der Vlueten *et al.,* 2015).

**Figure 1.**
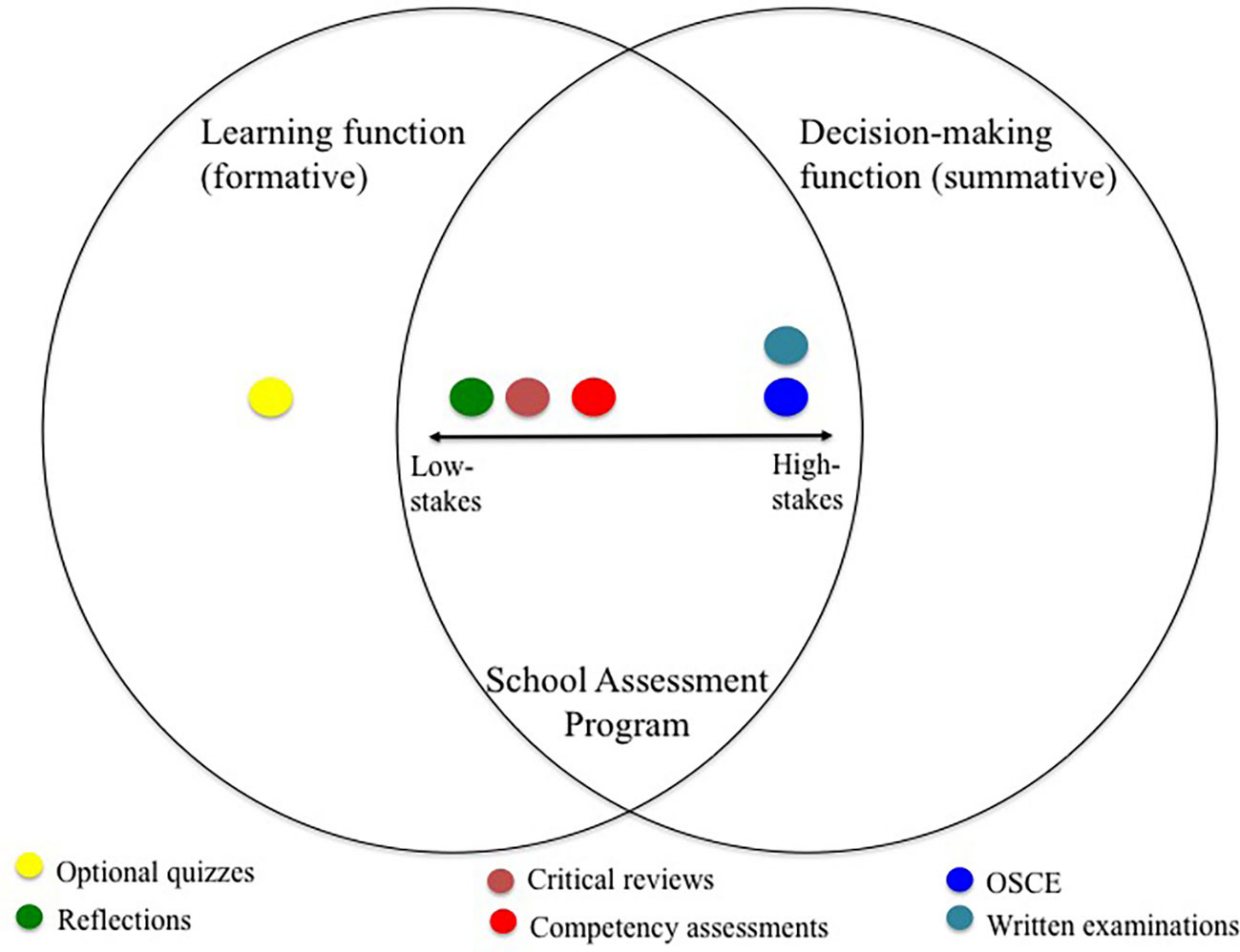
School assessment program combining the learning and decision-making functions of assessment.

Jessop *et al*. (2014, p.86) argue that assessment should be rebalanced from summative to formative in order to “engender learning for meaning, rather than for marks”. The School adopted an alternative approach by activating a set of continuous assessments that combined learning (formative) and decision-making (summative) functions. The model adopted is shown as Figure 1.

Figure 1 shows that the dichotomy between formative and summative is rejected in place of a system where assessments have both formative and summative dimensions. Summative assessment activities are summative inasmuch as they contribute to decision-making on student progress. That is, each assessment carries a certain weighting (percentage) towards the overall aggregate mark that is the basis to determine students’ progression. However, these activities also generate student feedback. Even high stakes end-of-year examinations result in feedback on performance tailored for every student. The only assessments that are purely formative (i.e. do not contribute to progression decisions) are optional assessments, for example, end-of-block quizzes that generate automated feedback. However, even these assessments can contribute to discussion in mentoring sessions.

To implement this model in the pre-clinical curriculum, 33 assessment activities were incorporated into an assessment matrix. These activities were presented to students through the learning portfolio and implemented across the academic year in first and second year. All were linked to existing learning outcomes in the curriculum and spread relatively evenly across the four Australian Medical Council (AMC) domains ( AMC Ltd, 2012) as follows: Science and scholarship (7 activities); Clinical practice (10 activities); Health and society (8 activities); and Professionalism and leadership (8 activities). Assessments include tasks that are set for students (reflection, research and critical composition) and observations of student skills and behaviours (competency assessments, skill credentialing).

The shift in assessment design described above represented an increase in continuous assessment that contributed to decision-making on student progress from 0% to 27.5% in first year and from 10% to 30% in second year. The School concurrently sought to build staff expertise in rubric development, providing written feedback aligned to these rubrics and embedding moderation processes into each assessment activity.

A six-year longitudinal research project was developed (2017-2022) to document and critically evaluate the changes as the School moved towards programmatic assessment. Schut *et al.* (2018, p.655) suggest an “urgent need” for this type of research. The overall long-term research question is: Does programmatic assessment offer a sustainable framework for educating and assessing students within a medical course? A subsidiary question, pertinent to this paper, is: To what extent has re-engineering continuous assessment in the learning portfolio supported the five principles of programmatic assessment?

This paper shares observations from the change process viewed through the lens of the continuous assessment program introduced in 2018. The impact of these changes on students and staff are described. The efficacy of the change process is discussed in relation to the key principles of programmatic assessment.

## Methods

The UNDA Human Research Ethics Committee (HREC) provided ethical approval to conduct the research, and a Research Team was formed in 2017. The team adopted action research as the framework to guide the conduct of the research, as the project aims to monitor, understand and influence the change process. Action research is the “systematic collection and analysis of data for the purpose of taking action and making change” ( Gillis and Jackson, 2002, p.264). It involves cycles of planning, implementing and observing and then reflecting on the processes and consequences of the action. As participants interact with the implementation in practice, its limitations and design constraints are identified by the researchers to refine and re-design subsequent solutions. Each cycle of the research runs over a one-year period so that planning, action, observation and reflection can inform the design of the next iteration of the change initiative. Mixed methods research, involving qualitative and quantitative methods, were used to build a depth of understanding.

Data were collected each year using questionnaires (students) and focus groups (staff). The student questionnaire included qualitative and quantitative components and posed two questions on the continuous assessment program. Students were asked to rate each of these statements according to a five-point Likert-type scale from Strongly Disagree (SD) to Strongly Agree (SA). For interpretation, a numerical value was assigned to each response from 1=SD to 5=SA. Quantitative data were downloaded from SurveyMonkey as Excel files for extraction of descriptive statistics and then imported into SPSS Version 25. All statistical procedures were conducted using SPSS Version 25.

To compare the responses from two independent groups to individual survey items (i.e. 2017 and 2018 students), a non-parametric statistic (median test) was used, as responses to individual survey items using a Likert-type response scale are ordinal in nature. The data is assumed to be independent as the study does not seek to compare matched pairs of data (i.e. the anonymity of student responses renders it impossible to match data from one year to the next). Summative aggregate scale scores from multiple items targeting the same construct, on the other hand, are interval data, therefore, a t-test was used for the comparison of mean scores between two independent groups.

Qualitative data were coded from students’ comments to open-ended questions on aspects of the learning portfolio that students felt were particularly beneficial to their learning journey and aspects that could be improved in the future. Qualitative data were also collected from staff through focus groups that were recorded and transcribed. These documents were imported into NVivo12 for coding and to facilitate a higher level of abstraction and explicitation of relevant themes and overarching ideas. Two researchers independently coded students’ comments and staff transcripts and then met to discuss and resolve differences in interpretation. Coding was then presented to the broader team in which ideas were further unpacked and themes developed using Braun and Clarke’s (2006) guide to thematic analysis.

## Results/Analysis

In 2017, 29% of the year 1 student cohort responded to the questionnaire (n=33) and in 2018, the response fraction across both Year 1 and Year 2 was 47% (n=98). For ease, the 2017 student cohort is described as Cohort 1 and the 2018 Student Cohort is described as Cohort 2. In 2017, 21 staff participated in focus groups. In 2018, 17 staff took part.

Two items from the student questionnaire were pertinent to the continuous assessment program and subsequent feedback. Table 1 and 2 compare student responses to the two statements on the following basis:

• over time in 2017 and 2018 within Cohort 1 ( Table 1); and
• for first year students - Cohort 1 2017 and Cohort 2 2018 ( Table 2).

For each table, median scores and the results of the Median Test are shown for each item. Table 1 compares Cohort 1 responses to items related to continuous assessment in the learning portfolio over time.

**Table 1.** Student Perceptions of Continuous Assessment Activities within Cohort 1 - Comparison of Median Scores (2017 and 2018)

Item	Median	Cohort 1 2017 (first year students) n=33		Cohort 1 2018 (second year students) n=51		
		> median	<= median	> median	<= median	Median Test ( *chi square (χ ^2^); df; p value*)
I enjoyed engaging with the activities in the learning portfolio	2	25	8	16	35	*χ*2 =15.797; *df*=1; *p*<0.001 ***
Excellent feedback was provided on activities	2	26	7	10	41	*χ*2 =28.653; *df*=1; *p*<0.001 ***

*** *Significant at p < 0.001 level*

Cohort 1 students enjoyed engaging with the portfolio activities more and had higher satisfaction with feedback in their first year than their second year. In addition to statistically significant differences between the 2017 and 2018 experience on items pertaining to the continuous assessment program, there is also a statistically significant difference in students’ perception towards the two statements (t=8.111; df=82, p<0.001). The aggregate mean rating which was positive in 2017 (M=3.18; SD=0.80; n=33) fell to a negative mean rating in 2018 (M=1.86; SD=0.69; n=51).

Table 2 compares first year students’ perceptions of items related to continuous assessment in the learning portfolio.

**Table 2.** First Year Student Perceptions of Continuous Assessment Activities - Comparison of Median Scores between Cohort 1 in 2017 and Cohort 2 in 2018

Item	Median	Cohort 1 2017 (first year students) n=33		Cohort 1 2018 (second year students) n=51		
		> median	<= median	> median	<= median	Median Test ( *chi square (χ ^2^); df; p value*)
I enjoyed engaging with the activities in the learning portfolio	3	10	23	11	36	χ ^2^ =0.477; df=1; p=0.490
Excellent feedback was provided on activities	2	26	7	13	34	χ ^2^ =20.285; df=1; p<0.001 ***

*** Significant at p < 0.001 level

In addition to a statistically significant difference between first year cohorts on the feedback provided, there is also a statistically significant difference in aggregate mean rating between the two cohorts of first year students in response to the two items on continuous assessment activities (t=4.103; df=78, p<0.001). The aggregate mean rating which was moderately positive for the first cohort of first year students in 2017 (M=3.18; SD=0.80; n=33) dropped significantly in the negative direction for the second cohort of first year students in 2018 (M=2.45; SD=0.78; n=47).

Overall, Tables 1 and 2 indicate that students had lukewarm perceptions of continuous assessment activities in the learning portfolio and feedback provided on these activities. There was a drop in both enjoyment with activities in the learning portfolio and feedback provided between 2017 and 2018. First year students in 2018 tended to be less satisfied with the feedback provided than first year students in 2017, and the mean score for second year students in 2018 to the item on feedback was the lowest mean response (1.63) to any of the items of the questionnaire.

Qualitative data was collected from students through the questionnaire and staff through focus groups (three focus groups for 21 staff in 2017 and three focus groups for 17 staff in 2018). The research team collated the qualitative data and reached consensus that: students generally felt that they were over-assessed; quality feedback was not provided in a timely manner; student choices were driven by extrinsic motivation; and there were significant gaps in students’ understanding of what constitutes studying medicine. Examples of student and staff comments, their relationship to the principles of programmatic assessment and the resultant themes, are provided in Table 3.

**Table 3.** Perceptions of Continuous Assessment Activities in 2018 - Qualitative Feedback

Student response category	Examples of comments	Relationship to principles of programmatic assessment	Theme
Perceived over-assessment	...less assignments. Workload is too high and the benefits do not correlate (Student 2018). The portfolio contributed significantly to my level of stress (Student 2018).	Continuous	Appropriate amount of assessment
Poor feedback practices	Need better feedback on written assignments. (Student 2018). More in-depth feedback on work, and returned in a timeframe that allows it to be relevant to our learning (Student 2018). Marking seemed thoughtless and halfhearted (Student 2018).	Continuous,Feedback-rich	Quality and timely feedback
Student choices being driven by extrinsic motivation	With the new continuous assessment policy the way it stands, you’re really making more work for students. Now they need not only to pass the multitude of assessment tasks, but they also have to pass each set of exams despite having less time available (Student 2018). The whole portfolio actually detracts from spending time learning content that is essential to clinical years (Student 2018). We have a mission as a school to create an assessment program that is more constructive and positive for learning, to change student perceptions. Currently the frame of mind is still very much around investment and grades. Until we can change the mindset... the stress will still be there (Staff 2018).	Coherent,Transparent	Stimulation of students’ intrinsic motivation
Poor understanding of what constitutes studying medicine	I have to spend so long writing reflections that there is little time to spend studying actual BCS and CCP material. The amount of reflective tasks could easily be halved and still have the desired effect. (Student 2018). The School is very clear that you have to pass the final assessment. Doing the continuous assessment takes time away from studying medicine is the way they see it. This is probably the source of the problem (Staff, 2018). When they talk about studying medicine they are talking about studying the science of medicine, there seems to be a lack of understanding about what medicine is. It has a caring component, a professional component, huge amount of communication and interpersonal skills. But they are obviously not placing much value on this. (Staff 2018).	Coherent,Transparent	Comprehensive articulation of the field of medicine through the assessment program

One of the aims of introducing the continuous assessment program was to facilitate multiple opportunities for learning via low-stakes data points whilst minimising the stress that arises from fewer high-stakes data points. However, the introduction of the program had the opposite effect with many students feeling that they were over-assessed, which increased their stress levels throughout the year. Generally students saw feedback as sub-standard considering it inconsistent and superficial. Student reactions to the introduction of the continuous assessment program reveals that their motivations are primarily extrinsic focussed on passing high-value assessment tasks. Students, at a relatively early stage of their training, also felt able to comment on what “real” medicine was, including the knowledge and skills that were going to be useful to them as they moved into clinical settings. Therefore, the key themes arising from the qualitative data are: develop an amount of assessment appropriate to the program, provide quality and timely student feedback, stimulate students’ intrinsic motivation for medicine, and comprehensively articulate of the field of medicine through the assessment program.

## Discussion

Wilkinson and Tweed (2019) propose that it is useful to view programmatic assessment as a framework to guide the quality improvement of assessment in general. This discussion focuses on how each of the principles of programmatic assessment was shaped by the continuous assessment program, and how the School’s incremental approach to the implementation of programmatic assessment was influenced as a result.

*Principle 1 (Continuous)* - Contemporary literature links continuous assessment with increased student engagement ( Holmes, 2015). However, the findings from the current study are unequivocal in reporting low levels of student enjoyment with continuous assessment activities. Qualitative data suggests that the source of student dissatisfaction was in the activities that were set for students (e.g. the number of reflections) rather than the School’s observations of student skills and behaviours (competency assessments, skill credentialing).

Finding an appropriate balance between the activities set for students and mid- and end-of-year examinations requires attention. In the pre-clinical years, the curriculum focuses on the basic clinical sciences (the BCS domain) and about 60% of assessments are targeted at BCS knowledge. The BCS domain provides only seven out of 33 continuous assessments in years 1 and 2 meaning that there is a heavy reliance on mid- and/or end-of-year examinations to cover BCS material. From a student’s perspective, examinations are the focus with continuous assessments viewed as an unwelcome distraction. To optimise ongoing opportunities for learning, this imbalance requires attention through more frequent testing and feedback in the BCS domain. Current mid- and end-of-year examinations could be broken down into smaller, targeted tests, perhaps coupled with progress testing - a longitudinal assessment strategy based on the growth trajectory of students’ knowledge ( Schuwirth and van der Vlueten, 2012). Certainly a greater number of lower stakes knowledge-based assessments are required if the School is to move further towards programmatic assessment.

*Principle 2 (Feedback-rich) -* Students were generally critical of the quality and timeliness of feedback provided for assessment tasks in the continuous assessment program. Re-engineering continuous assessment created pressures on staff to develop more sophisticated rubrics, implement rigorous moderation processes to enhance the defensibility of assessment results, and provide quality and timely written feedback to students. Osuala *et al.* (2018) recommend that academic staff are trained in implementing continuous assessment, particularly in providing feedback as this is said to have a direct impact on student performance. A targeted professional development program, crystalising current thinking on feedback ( Boud and Molloy, 2013), and emphasising the importance of quality and timely dialogic feedback with students, is required.

*Principle 3 (Mentored)* - Data-informed mentoring is dependent on the data collected, shared and synthesised by students with the assistance of their mentor. Findings suggest that students were overwhelmed by the number of continuous assessments. The implementation of the continuous assessment program also did not coincide with a concurrent reduction in the stakes attached to examinations. From a student’s point of view, this increased anxiety. Schut *et al.* (2018) warn of the dangers of assessment overload and to some extent the School may have been guilty of this, but with a devolved curriculum based upon domains, it is unsurprising that most domains sought to optimise the impact of their curricula by seeking to ensure that a large number of learning outcomes for which they were responsible were assessed. Jessop *et al.* (2014, p.86) urge curriculum designers to “create connections, sequences, timing and logical flow of assessment tasks across the whole programme.” The current assessment program at the School encourages domains to compete for students’ attention. Strong leadership is required to better integrate assessment tasks across domains and ensure that the assessment program as a whole does not become unnecessarily inflated. Such integration might also address student concerns that some assessment activities (particularly reflective activities) were repetitive and formulaic.

*Principle 4 (Coherent)*- Findings revealed that students, at an early stage of their medical training, exhibited strong beliefs about what constitutes medicine. These beliefs tended to prioritise clinical and scientific knowledge and skills over the challenging and confronting aspects of medicine for which there are no “right answers.” Curricula that deal with, for example, ethical and social dilemmas, boundaries and professionalism, and even students’ own personal wellbeing in the context of a demanding work environment, were seen by students as peripheral to “real medicine”. Requirements for bigger picture understandings of health and society, standards of professionalism, or leadership conceptions in the health professions, represented in the continuous assessment program, were given a lower priority.

Students are right to value the science of medicine, but struggle to see where other important domains fit, often until after graduation. The School sought to confront this lack of understanding by situating a set of continuous assessments alongside examinations to arrive at more rounded and holistic decision-making on student progression. However, because decisions on student progress are determined by adding marks achieved in each assessment, the School has left itself open to what Frank *et al.* describe as *compensation* (2010, p.640) or “the phenomenon of allowing ability in one essential domain (e.g., procedural skills) to compensate for lack of ability in another (e.g., communication).” With the assessment balance skewed towards examinations focused on scientific knowledge, the School is affirming students’ existing, and somewhat naïve, beliefs about medicine.

Addressing compensation requires explicit connections to be made between the curriculum and the competencies that students are required to demonstrate on graduation. The AMC Limited (2012) gauges the quality of medical schools on how science and scholarship, clinical practice, professionalism and leadership, and health and society are integrated into the curriculum. Better articulating the importance of AMC domains to “collectively provide the requirements that students must demonstrate at graduation” ( AMC, 2012, p.3) is a priority for the School. In programmatic assessment, data points are mapped to an overarching structure (e.g., competency framework) so that students can understand the value of what they learn ( van der Vlueten *et al.,* 2015). Explicit signposting and mapping of assessment items to AMC domains would help to ensure the coherency of assessment.

*Principle 5 (Transparent)* - Student sentiments on continuous assessment program reveals a dissonance between the School’s vision for assessment and how this vision is interpreted. Incongruity between designers’ intentions and learners’ perceptions was also noted in a study on the implementation of programmatic assessment by Schut *et al.* (2018). The authors (2018, p.660) saw the provision of grades as being at the root of the problem suggesting that providing grades risks students focusing on “outcomes and competition rather than stimulating a focus on continuous improvement”. Although the continuous assessment program at the School caused students to engage more thoughtfully with assessment activities, it also increased students’ anxiety levels and further fuelled strategic learning. Extrinsic motivation (i.e. the focus on gaining marks) is the antithesis of the assessment program that the School is aiming for, and yet staff perceived that this form of motivation was widespread.

Pereira *et al.,* (2018, p.563) believe that there is “substantial evidence that our current reliance on standardized tests and grades has little, if any, correlation with clinical performance.” One way of changing students’ pre-occupation with grades rather than learning, is to credential students on a pass/fail basis as opposed to employing grading schemas. This change would lessen student competitive behaviours that are arguably inappropriate for future workplaces.

Programmatic assessment requires expert panels to be formed to make judgments on student progress ( van der Vlueten *et al.,* 2015) assigning no weighting to individual assessment tasks. The final grading is done subjectively using overarching assessment rubrics, linked to a competency framework, based on all available assessment information. This type of holistic judgement may ultimately be more trustworthy than basing progression decisions on numerical scores denoting success in an examination or a collection of unrelated continuous assessments. One of the challenges facing advocates of programmatic assessment, however, is to persuade stakeholders that making holistic judgments is legitimate. This challenge includes developing auditable processes so that decisions are transparent and defensible.

There are limitations to the current study. It is focused only on the preclinical medical program and does not offer any sense of whether the changes adopted will have any longer term impacts, particularly after students graduate. The study ran at one University and although it will ultimately cover a six-year timeframe, findings should be gauged within the context of the setting in which the research was situated. Relatively low response rates were noted. The questionnaire used in the study contained only two questions on the continuous assessment program. To gain a more nuanced understanding of the impact of this program, a more sophisticated questionnaire is required that probes the different components of the continuous assessment program along with its perceived integration with other forms of assessment, and how it relates to student motivation levels and anxiety.

## Conclusion

This paper has attempted to explain the trajectory of the School’s journey towards programmatic assessment. Changing conceptions around formative and summative continuous assessments have exposed tensions in the assessment program, serving to fragment it, and fuel strategic learning amongst students. Focusing on how curriculum change has impacted on the five principles of programmatic assessment is a useful way to identify and address tensions that arise, and helps create a more robust assessment system where components are in harmony rather than antagonistic.

## Take Home Messages

• Balancing activities set for students in a learning portfolio with frequent knowledge-based testing will relieve pressure on examinations.
• A move towards programmatic assessment requires complementary professional development emphasising the importance of quality and timely dialogic feedback with students.
• Integrating assessment tasks across learning domains and ensuring that an assessment program does not become unnecessarily inflated requires strong leadership.
• Explicit signposting and mapping of assessment items to a competency framework is a useful first step in ensuring that assessment is coherent to students.
• The assessment program should encourage students to value the learning journey over the grades that evidence the learning journey.

## Notes On Contributors

Associate Professor Frank Bate is the Director of Medical and Health Professional Education in the School of Medicine at the University of Notre Dame Australia, Fremantle, Australia.

Professor Sue Fyfe is an adjunct professor with the Faculty of Health, Curtin University of Technology, Perth, Australia.

Dr Dylan Griffiths is the Quality Assurance Manager in the School of Medicine at the University of Notre Dame Australia, Fremantle, Australia.

Associate Professor Kylie Russell is a curriculum developer in the School of Medicine at the University of Notre Dame Australia, Fremantle, Australia.

Associate Professor Chris Skinner is the Chair of the Personal and Professional Development Domain in the School of Medicine at the University of Notre Dame Australia, Fremantle, Australia.

Associate Professor Elina Tor is a psychometrician in the School of Medicine at the University of Notre Dame Australia, Fremantle, Australia.

## Declarations

The author has declared that there are no conflicts of interest.

## Ethics Statement

University of Notre Dame Australia. Unconditional HREC approval 017066F.

## External Funding

This article has not had any External Funding
