# Supplementary material for: Does an incremental approach to implementing programmatic assessment work? Reflections on the change process
Source: MedEdPublish (2016). 2020 Mar 27;9:55. [Version 1] doi: 10.15694/mep.2020.000055.1 (PMC10697442; doi:10.15694/mep.2020.000055.1)
Supplement: Peer review history

# Reviewer report: Reviewer response for version 1

**DOI:** 10.21956/mep.19997.r30457

**Published:** 2020-04-19

**Authors:** Balakrishnan Nair

**Affiliations:** 1 Centre for Medical Professional Development and University of Newcastle

This review has been migrated. The reviewer awarded 4 stars out of 5 I enjoyed reading this article. More and more schools and Colleges are trying to introduce competency-based education and programmatic assessment.Assessment should be continuous, formative with the ongoing feedback and mentorship, to prepare the graduates for the future workforce. So this action research is timely, and some of the lessons are generalisable. It is interesting to see the feedback from the faculty about lack of assessments in the so-called “soft skills “ like professionalism, communication and teamwork. From my own experience in running a workplace-based evaluation, it is not the tools, which are crucial; the challenge is assessors training and calibration, and learner buy-in. It will be interesting to see further evidence from this action-based research. It may be a few years before we can know the outcome-based data from programmatic assessment graduates.

Reviewer Expertise:

NA

No decision status is available

# Reviewer report: Reviewer response for version 1

**DOI:** 10.21956/mep.19997.r30459

**Published:** 2020-03-29

**Authors:** P Ravi Shankar

**Affiliations:** 1 American International Medical University

This review has been migrated. The reviewer awarded 5 stars out of 5 I read with great interest this article on programmatic assessment at an Australian medical school. The authors have clearly explained the important concepts behind programmatic assessment. They have described the methodology of the study well. They have highlighted the limitations of the study. The low response rate and the simple nature of the quantitative part of the questionnaire which had only two questions were among them. I think a lot of rich information was obtained from the qualitative responses. I am stuck by the dichotomy in perception between the developers of the assessment and the students. The developers intended it to be a kind of regular gentle monitoring and signposting for the students to guide them in the right direction and help reduce student stress. But thinking back to my student days I guess every exam is an exam and if something is going to be assessed then there is always stress associated with it. As a student being examined all the time is going to be tough. Students were of the opinion of not being provided adequate and good quality feedback. They also felt there was too much of reflection and the basic sciences were getting less priority. The results of the study raise some important questions for the authors and for further development of the curriculum and of programmatic assessment at their medical school. It also raises important questions about programmatic assessment and the need for further information and clarifications as the method become more popular. This is an important article of broad interest to educators.

Reviewer Expertise:

NA

No decision status is available

# Reviewer report: Reviewer response for version 1

**DOI:** 10.21956/mep.19997.r30458

**Published:** 2020-03-27

**Authors:** Felix Silwimba

**Affiliations:** 1 University of Lusaka

This review has been migrated. The reviewer awarded 5 stars out of 5 this study is informative on students perceptions of assessments.

Reviewer Expertise:

NA

No decision status is available
